# Gigantic blue shift of two-photon–induced photoluminescence of interpenetrated metal–organic framework (MOF)

**DOI:** 10.1515/nanoph-2023-0383

**Published:** 2023-09-12

**Authors:** Zhihui Chen, Defeng Xu, Menglong Zhu, Yueting Wang, Junfan Feng, Chuancun Shu, Si Xiao, Jianqiao Meng, Jun He

**Affiliations:** Hunan Key Laboratory of Nanophotonics and Devices, School of Physics and Electronics, Central South University, Changsha 410083, China; Department of Applied Physics, School of Microelectronics and Physics, Hunan University of Technology and Business, Changsha 410205, China

**Keywords:** two-photon photoluminescence, blue shift, metal–organic framework

## Abstract

As an important means of modern science and technology, multiphoton fluorescence plays an essential role in high-resolution imaging, photochemistry, micro- and nano-processing and clinical diagnosis. Multiphoton fluorescence usually shares the same radiative channel as its intrinsic fluorescence. Under multiphoton excitation, except for red shift fluorescence caused by the reabsorption effect, gigantic blue shift of multiphoton fluorescence is rarely reported. In this work, metal–organic frameworks (MOFs) with 7-fold and 8-fold interpenetration are successfully synthesized. The synthesized 8-fold interpenetrated MOFs show unexpectedly giant blue-shifted (∼40 nm) two-photon–induced fluorescence compared with its fluorescence emission. Specific optical selection rules lead to different final transition states in one-photon absorption and two-photon absorption. The density functional theory (DFT) and time-dependent density functional theory (TDDFT) simulations show that, under two-photon excitation, electrons and holes can be more delocalized, and intermolecular interactions mainly govern the emission process of 8-fold interpenetrated MOFs. Highly excited electronic states of the interpenetrated MOFs are effectively excited and emitted under two-photon excitation, thus generating the inevitable blue-shifted two-photon–induced fluorescence emission. Our work provides a guide for exploring the excitation mechanism of fluorescent MOFs and offers an access to a tunable all-optical single-crystal device.

## 1 Introduction

Nanomaterials with highly efficient nonlinear optical (NLO) responses are in great demand for applications in all-optical optoelectronics, bio-photonics, information processing and the like [1–3]. Among the NLO-active materials, multiphoton luminescent material has always been a research hotspot due to its application in photodynamic therapy, biological imaging and sensing, optical data storage and nano-processing, etc. [4–8]. During the multiphoton process, materials could simultaneously absorb two or more photons in the near-infrared (NIR) window, and the electrons were stimulated from the ground state to the excited state. The emitted photons present frequency greater than those of the incident photons, namely, producing emission at shorter wavelength [9–12].

Compared with traditional NLO-active materials, metal–organic frameworks (MOFs) show the advantages of enhanced stability, tunability of both metal ions and organic ligand, easily decorated nano-channels and large capacity, which endows MOFs excellent optical behaviour [13–26]. Superior linear optical properties guarantee efficient NLO response due to the substantial oscillator strength and hyperpolarizability [27]. For the pursuit of multi-function and intelligent response of MOFs, increasing research attention has been paid to rationally design luminous MOFs by incorporation of organic photoactive species as organic linkers [28–32], by appropriate functionalization of the linker [33–35] or by orderly arrangement luminophores in crystalline frameworks [36–40], which could specifically regulate their physical properties such as band gap, optical properties, exciton delocalization, migration and lifetime [41–44]. Beyond that, the structural variation and ordered arrangement of the frameworks also brings new opportunities to regulate the photo-physical behaviour of MOFs [16, 45–48]. Vittal et al., reported that the second harmonic generation (SHG) and two-photon photoluminescence (2PPL) of Zn MOFs could be greatly enhanced through the structural transformation from 7- to 8-fold interpenetration. Accompanied by the increase of interpenetration, the π–π interaction between the individual diamondoid networks (dia net) is strengthened, which ultimately boosts the transition dipole moment and NLO conversion efficiency of the interpenetrated MOFs [48, 49]. Qian et al., extended their research on the correlation between the structural variation and the corresponding NLO properties of MOFs. They synthesized two europium MOFs (two-dimensional (2D) ZJU-23-Eu and 3D non-centrosymmetric ZJU-24-Eu) with different coordination modes by carefully regulating solvent contents. The variation in both structural symmetry and spatial connectivity resulted in the changes in SHG, 2PPL and three-photon photoluminescence (3PPL) responses.

Most fields view luminescent MOFs as merely polymer-like platforms with highly efficient structure-related linear and nonlinear optical properties and overlook the unique features of MOFs and NLO interaction mechanism that distinguishes them from the linear optical process [44]. Multiphoton absorption (MPA) properties of MOFs are strongly connected with the photophysical properties of the diverse electronic states, e.g., inter- or intra-ligand interactions, metal centre emissive states and metal–ligand interactions, which promote long-live charge transfer. Since the crystalline nature and the fact that MOFs are constructed by organic chromophore linkers, the existence of excitons in MOFs is also proposed [50–52]. However, the characterization of excited and excitonic states of MOFs is rarely reported due to the difficulties of high-quality sample preparation, spectroscopic characterization technique and high cost of computation [53–56]. Nevertheless, multi-photon excited electronic state properties and the distinct light–matter interaction mechanism of multi-photon process, in the molecular-like system, MOFs, are essential factors in the correct description of multi-photon absorption and emission processes.

In this work, Zn (II) MOFs were successfully synthesized by using trans-2-(4-pyridyl)-4-vinylbenzoate (pvb) ligand under different experimental conditions. The isolated MOFs, [Zn(pvb)_2_]·DMF (**1**) and [Zn(pvb)_2_] (**2**), respectively, possess 7-fold and 8-fold interpenetrations. Systematic investigation of two-photon emission and the corresponding dynamic properties of Zn (II) MOFs have been conducted via ultrafast time-resolved fluorescence spectroscopy (TRPL) and time-resolved emission spectroscopy (TRES). Surprisingly, the 2PPL emission of 8-fold Zn MOFs was strongly blue-shifted (∼40 nm) with respect to its one-photon photoluminescence (PL) emission, while for 7-fold interpenetrated MOFs, the emission profiles for 2PPL only showed a minor blue shift (∼4 nm) compared with that of PL. Such a gigantic blue shift of 2PPL compared with PL in MOFs is the first time observed. The dynamics processes of the gigantic blue shift of 2PPL were carefully analysed, which reveals that a two-state model dominated the emission process. The experimental results were confirmed by density functional theory (DFT) and time-dependent density functional theory (TDDFT) calculations that the effective excitation and efficient emission of highly ionization states under two-photon excitation, besides the lowest energy vibrational state, induced the huge blue shift of the 2PPL compared with PL emissions. Our results provide a new perspective for interpretation of the luminescence mechanism and nonlinear optical properties of MOFs, which would promote the development of nonlinear optical single crystal devices.

## 2 Results and discussion

### 2.1 Synthesis of [Zn(pvb)_2_]·DMF (1) and [Zn(pvb)_2_] (2)

**Figure 1:**
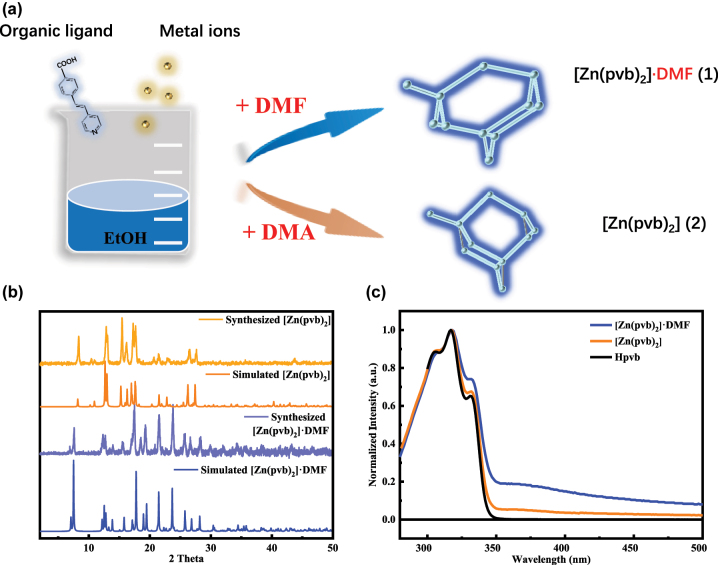
The schematic diagram of synthesis process**, **the powder XRD patterns and absorption spectra. (a) Schematic diagram of synthesizing **1** and **2** microcrystals. (b) The corresponding powder XRD patterns of **1** and **2** microcrystals. (c) Absorption spectra of **1**, **2** microcrystals and Hpvb ligand.

By utilizing Hpvb organic linker under different experiment conditions, we can obtain [Zn(pvb)_2_]·DMF (**1**) and [Zn(pvb)_2_] (**2**) with 7-fold and 8-fold interpenetrations, respectively (Figure 1(a and b)). The synthesis of compound **1** and **2** was reported in our previous literature [48]. Crystal structure of **1** belonged to non-centrosymmetric monoclinic space group *C*_*c*_ [57]. The building blocks are made up of two O atoms of a chelating carboxylate group, two pyridyl N atoms and an O atom from a monodentate carboxylate group, with a distorted five-coordinate geometry (Figure S1). The tetrahedral Zn^II^ node expends and constructs the framework into a diamondoid framework with 7-fold interpenetration. DMF molecules serve as guest solvent in the crystal structure [48].

The synthetic solvent of compound **2** is a mixture of DMA and water. There is no guest solvent molecule retained in the crystal lattice. The two crystallographically distinct Zn^II^ atoms are located on the 2-fold axes and coordinate with two pyridyl ligands and two carboxylate groups. Zn1 has a distorted octahedral geometry with two pyridine nitrogen atoms and two chelating carboxylates, whereas Zn2 coordinates with both coordinating carboxylates in monodentate mode resulting in a tetrahedral geometry (Figure S2). This dia net exhibits a more compact 8-fold interpenetrated network along the crystallographic *b*-axis with no guest solvent approachable space [48, 57].

Thermogravimetry (TG) analysis of **1** shows a slowly weight loss of DMF guest solvent from room temperature and a rapid weight loss from 110 to 210 °C. Total weight loss (experimental: ∼13.0 %, calculated: 12.5 %) indicates the removal of DMF guest (Figure S3). Interestingly, the 7-fold interpenetrated MOF **1** could transform to the 8-fold interpenetrate MOF **2** only through the desolvation process in a SCSC (single-crystal-to-single-crystal) manner. The detailed mechanism of structure transformation was reported in our previous article [48]. During the process, the guest DMF molecule was removed from the structure of **1** along *c*-axis, and then the adjacent nets came close to each other to fill the void. The bonds between Zn centres and organic ligands were broken and reformed. The interpenetration increased from 7-fold MOFs **1** to 8-fold MOF **2**, accompanied by the significant geometry change of the dia net. The experimental diffraction patterns of **1** and **2** matched well with the simulated diffraction patterns of the 7-fold and 8-fold interpenetrated MOFs (Figure 1(b)).

### 2.2 Linear optical properties of 1 and 2

Linear optical properties of Hpvb ligand revealed main absorption peaks around ∼305, 317 and 332 nm, which dominated the absorption of **1** and **2** (Figure 1(c)). The ground microcrystals of **1** and **2** were dispersed in DMF to measure absorption spectra. Both **1** and **2** showed broad absorption tails in the visible window, respectively (Figure 1(c)). These broad absorption bands of **1** and **2** were ascribed to the metal–ligand interactions since the closed shell (d^10^) Zn (II) ions could not contribute to the absorption in the visible region. Upon 340 nm excitation, the emission profile of Hpvb displayed a typical mirror image of its absorption spectrum with multiple emission bands (Figure S4). The emission bands of Hpvb were analysed by fitting with Gaussian function with the main peak at 437 nm and shoulder peaks at 408, 432 and 477 nm (Figure S5(a)). Single crystals of **1** and **2** showed emission bands peaked at 458 and 470 nm, respectively, which can be probably assign to ligand-dominated fluorescence emissions since weakly similar emissions were also observed with Hpvb ligand [57]. Additionally, we conducted PL excitation (PLE) measurement for **1** and **2** recorded at respective PL peaks (458 nm for **1** and 470 nm for **2**). PLE spectra for both **1** and **2** showed broad excitation band, multiple shoulder peaks at short wavelength side and a main peak at long wavelength side, respectively. Compared with their respective linear absorption spectra, the main excitation peaks located at the position of the absorption tail of the linear absorption spectrum, corresponding to the electron transition process caused by metal–ligand interactions (Figure S5(b and c)). Additionally, single crystal of **2** presented 10 nm red-shifted PL compared to **1** due to the increased π–π interactions between each dia net along with increased interpenetration degree.

### 2.3 Giant blue shifted two-photon photoluminescence

**Figure 2:**
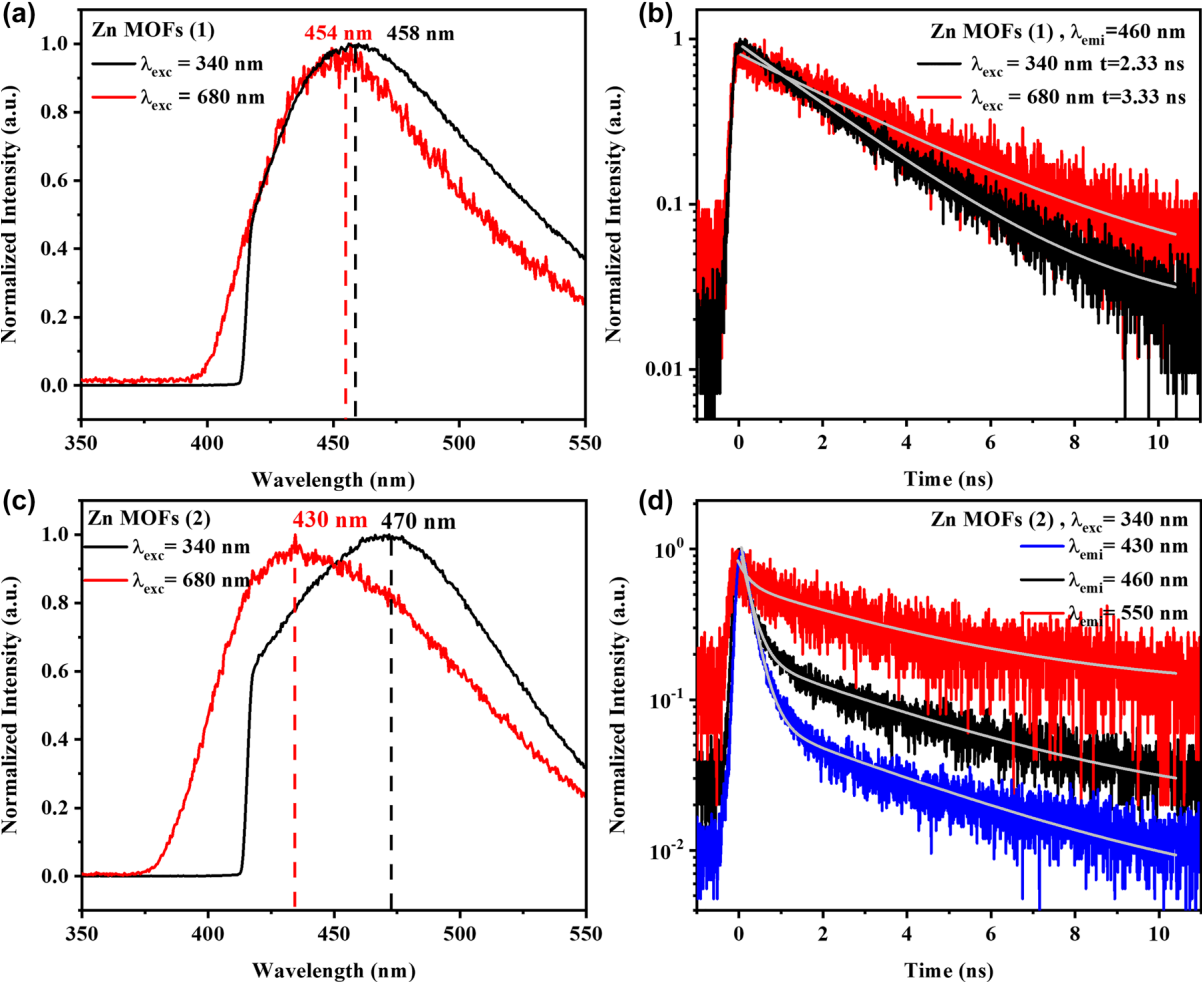
PL, 2PPL emission spectra and the corresponding dynamics of **1** and **2**. (a) PL and 2PPL emission spectra of **1** under 340 and 680 nm, respectively. (b) PL and 2PPL dynamics of **1** with a collection wavelength of 460 nm. (c) PL and 2PPL emission spectra of **2** under 340 and 680 nm, respectively. (d) PL dynamics of **2** with collection wavelengths of 430, 460 and 550 nm.

NLO studies on **1** and **2** single crystals were investigated under a microscope upon excitation of near infrared (NIR) femtosecond laser. The corresponding NLO signals were collected in reflection geometry via an inverted microscope (Figure S6, detailed experiment setup can be found in Experimental section). Generally, the same fluorescence emission spectrum would be observed irrespective of the excitation wavelength, known as Kasha’s rule. The 2PPL spectra of Hpvb ligand was identical to its PL spectra (Figure S4(a)) which indicated that their single-photon excited emissions shared the same radiation channel as that of two-photon excitation emissions. The resorption effect is always observed in single crystals upon two-photon excitation, where the emitted photons can be re-absorbed and re-emitted multiple times within the single crystals and increased penetration depth, resulting in red-shifted PL emissions [58]. In order to determine the optimal two-photon excitation wavelength, we conducted the two-photon photoluminescence excitation (2PLE) measurements for **1** and **2** in the wavelength range of 700–890 nm. As the wavelength decreased, the 2PLE intensities of **1** and **2** increased (Figure S5), which was consistent with respective linear absorption spectra. It can be inferred that with the further reduction of excitation wavelength, the 2PLE intensity will enhance for both **1** and **2** single crystals. Therefore, the relevant 2PPL studies of **1** and **2** are measured under 680 nm excitation, the minimum excitation wavelength of our laser system. Differently, the 2PPL of **1** peaked at 454 nm, which was ∼4 nm blue shift compared with its PL emission (Figure 2(a)). Surprisingly, the 2PPL emission of **2** brings out a giant blue shift (∼40 nm) compared with its PL emission. Such a large, blue-shifted emission induced by MPA is the first time observed in MOFs. Additionally, excitation power dependence of the multi-photon PL signals displayed quadratic power dependence for **1** and **2** under 680 nm excitation, validating 2PPL (Figure S7). Steady-state PL (*λ*_exc_ = 340 nm) and 2PPL (*λ*_exc_ = 680 nm) spectra of 2 were also conducted under 77 K (Figure S8). As the temperature down to 77 K, the peak positions of both PL (∼469 nm) and 2PPL (∼434 nm) of **2** were nearly unchanged compared with that measured under room temperature (RT). Therefore, the major radiative channels are dominated by the emission from ligand under both RT and 77 K conditions. Besides, the spectral width of PL and 2PPL under 77 K became narrower than that measured under RT due to the suppression of exciton (electron)–phonon interaction under low temperature [59, 60].

To investigate the relaxation processes of **1** and **2** in detail, time-resolved PL (TRPL) measurements were conducted to determine the average time of the molecule spent in the excited state prior to returning to the ground state. Under 340 nm excitation, the emission of **1** occurred with a typical PL lifetime of ∼2.33 ns, resulting from a thermally equilibrated excited state, that is, the lowest energy vibrational state (Figure 2(b), black line). A similar dynamic process of **1** under two-photon excitation was also observed with a lifetime of ∼3.33 ns. As expected, the distinctive crystal structures of **1** and **2** should induce different linear and nonlinear optical properties. The PL lifetime of 2 presented an average lifetime of ∼3.21 ns (*λ*_exc_ = 340 nm, *λ*_emi_ = 460 nm) composed of two constituents: $A_{11}^{PL}=$ 23 %, $\tau_{11}^{PL}=$ 0.25 ns; $A_{12}^{PL}=$ 77 %, $\tau_{12}^{PL}=$ 4.1 ns. As the collection wavelength increased from 430 nm to 550 nm, the corresponding PL lifetime expanded from ∼2.31 ns to ∼3.98 ns, which indicated that the fluorophores partially existed in other ionization states, besides the lowest energy vibrational state [61]. A similar situation also occurred for **2** under two-photon excitation (Figure S9). The detailed fitting parameters of TRPL curves of **2** upon one and two-photon excitation were summarized in Table S1, respectively.

**Figure 3:**
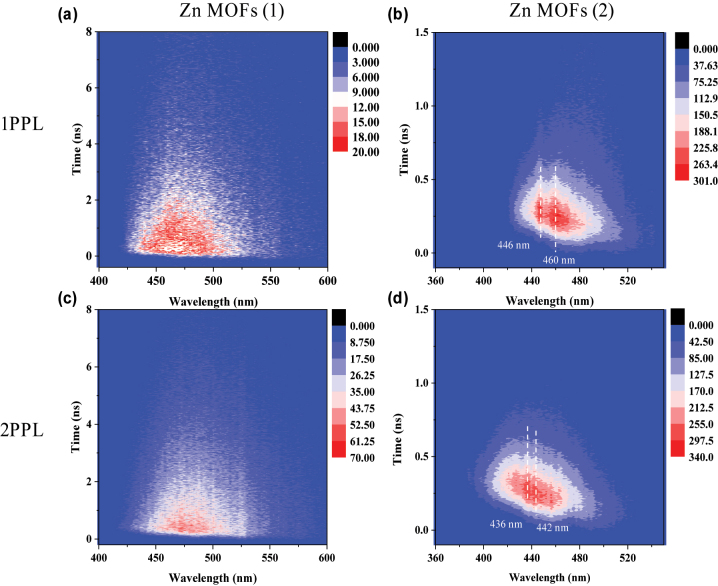
TRES images of **1** upon one-photon excitation (1 PE) (*λ*_exc_ = 340 nm) (a) and 2 PE (*λ*_exc_ = 680 nm) (c); TRES images of **2** upon 1 PE (*λ*_exc_ = 340 nm) (b) and 2 PE (*λ*_exc_ = 680 nm) (d).

To further visualize the dynamic processes of PL and 2PPL emissions of **1** and **2**, time-resolved emission spectrum (TRES) was performed, which could provide the information about how emission spectra can be affected during the excited-state lifetime. Classification from spectral properties, TRES spectra are commonly divided into two categories: continuous model and a two-state model [61]. In continuous model, the PL spectra do not change with time. While for the two-state model, the emission spectrum is composed of distinct emission profiles generated from two states of the fluorophore, typically separated by an energy barrier. The wavelength dispersion and spectral shape of PL and 2PPL spectra of **1** were nearly unchanged with time evolution (Figure 3(a and c)), which conformed to the continuous spectral shift model, suggesting the emissions occurring from the same state. While for single crystal **2**, TRES spectra for both PL and 2PPL displayed complex time-dependent decays. Under 340 nm excitation, TRES image revealed two primary emission centres (*λ*_max_ = 446 and 460 nm), while upon 680 nm excitation, emission centres blue shifted to *λ*_max_ = 436 and 442 nm, correspondingly (Figure 3(b and d)). The dynamics of the emission species depended on the observation wavelength, which was consistent with the results of TRPL measurement of **2** as shown in Figure 2(d) and Figure S9.

### 2.4 Density functional theory (DFT) and time-dependent density functional theory (TDDFT) calculation

To gain a better understanding of the giant blue shifted 2PPL of **2**, theoretical calculations including the geometry optimizations and vibration frequency calculations were carried out in Gaussian16 package [62], and meanwhile, DFT [63] and TDDFT [64] were employed for the calculations of the ground states and singlet excited states, respectively. The optimized structures of ground states were further employed to process the electron excitation calculations, and a total of 50 excited states were considered. In addition to the absorption and emission spectra, charge transfer matrix (CTM) analysis from ground state to excited state, the electron–hole analysis from ground state to excited state, the transition density matrix (TDM) between excited states, as well as the differential charge density (DCD) analysis between excited states were also constructed to clarify the differences between the processes of 1 PE and 2 PE of **2** (detailed calculation methods can be found in Supplementary Information).

Figure S10(a and b) displays one-photon (two-photon) absorption spectrum of **2**, where the left and right axes represent the molar absorption coefficient and oscillator strength (two-photon absorption cross section), respectively. The calculated one-photon absorption (1 PA) curve consisted of three main transitions (S0 → S19, S0 → S20, S0 → S27) and two feeble electron transitions (S0 → S23, S0 → S30), which together produced two characteristic absorption bands around ∼300 and 335 nm. CTM maps and distribution of electrons and holes clearly showed that the charge transfer process predominately happened between the benzoic acid group and pyridyl group within a single Hpvb bridge (Figure S12). Typically, two-photon absorption (2 PA) involves two transition processes, which excite a molecule from the ground state to a higher intermediate state and from the intermediate state to an excited state simultaneously. If the two transitions possess considerable transition probability and transition dipole moments, the 2 PA process becomes significant. Under 2 PE, the simulated absorption spectrum composes two main transitions and a subsidiary peak located around ∼596, 668 and 683 nm (Figure S10(b)). The difference between the profiles of one- and two-photon absorption spectrum may be due to their obeying different optical selection rules [65, 66]. Since the photon has the odd parity, in the centrosymmetric system, one- and two-photon transitions exclude each other: one-photon transitions occur between states with different parity, while two-photon transitions occur between states with the same parity. In non-centrosymmetric systems, such as **1** and **2** with space groups of *C*_*c*_ and *C*_2_ symmetry, parity is not a good quantum number and there are some transitions that are allowed for both one- and two-photon absorption. Nevertheless, the oscillator strengths of electronic states are generally different between one- and two-photon processes [67, 68]. Table S2 summarizes the corresponding main transition channels of 2 PA obtained from **2**. Under 680 nm excitation, the transition of S0 → S23 and S0 → S24 predominately governed the 2 PA process. Both TDM and DCD maps revealed the larger separation degree of the electron and hole in 2 PA than that of 1 PA process of **2**. The charge transfer processes occurred between the alternative organic chains accompanied by 2 PA process (Figure S13).

**Figure 4:**
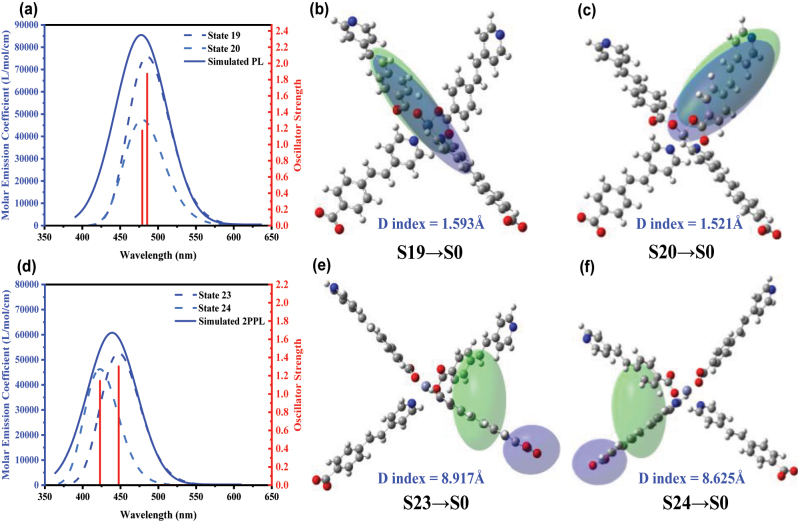
The simulated emission spectra and charge distributions of **2** under one-photon and two-photon excitation. (a) Emission spectra of **2** under one-photon excitation condition, left axis: molar emission coefficient; (b and c) distribution of electron (coloured by green ellipse) and hole (coloured by blue ellipse) of S19 → S0 transition, S20 → S0 transition; (d) emission spectra of **2** under two-photon excitation condition. (e and f) Distribution of electron (coloured by green ellipse) and hole (coloured by blue ellipse) of S23 → S0 transition, and S24 → S0 transition.

Figure 4(a)–(d) indicated the simulated one- and two-photon emission spectrum of **2**. Under one-photon excitation, two emission species (S19 → S0, S20 → S0) highly overlapped in the spectrum. The overall PL of **2** exhibited as localized emissions (LE) within a molecule peaked at ∼477 nm, which was consistent with the experiment result ( $\lambda_{PL}^{\exp}=470\;\;\text{nm}$ ). These two emission species have different radiation channels. Thus, the overall emission dynamics would exhibit a bi-exponential PL decay, which was consistent with measured TRPL results of **2** (Figures 2(d) and 3(b)). *D* index was used to evaluate the separation degree of the electron and hole. Under 2 PE, the two distinctive emissive states (S23 → S0; S24 → S0) showed comparable oscillator strength with each other. Such dual emission centres nature is achievable when energy and electron/charge transfer between the two emissive species are extremely suppressed [69, 70]. The calculated ensemble emission bands moved toward the shorter wavelength and generated overall 2PPL around ∼438 nm. The larger *D*-index of 2PPL compared with PL was owing to the intermolecular charge transfer (ICT) involved in the respective emission states.

**Figure 5:**
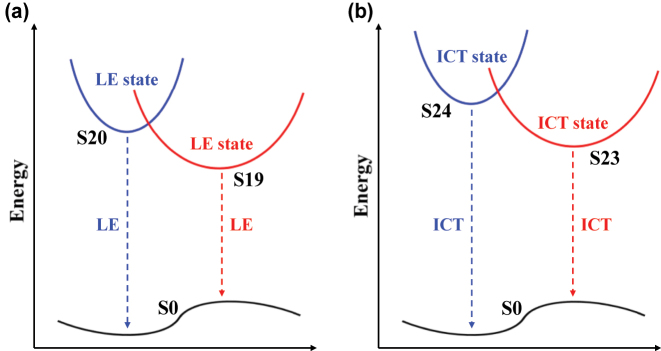
Schematic diagram of calculated emission of **2** under (a) one- and (b) two-photon excitation condition.

Figure 5 shows the schematic diagram of calculated emission of **2** under one- and two-photon excitation conditions. Optical selection rules are crucial in interpretating of atomic and molecular spectra which can be easily understood by applying angular momentum conservation and parity conservation parameters to absorption process of **2**, upon one- and two-photon excitation, the transitions occur between different electronic states obeying distinctive optical selection rules. According to the Strickler–Berg equation *k*_*r*_ = 0.688 ${\left\langle \tilde{v}\right\rangle }_{av}^{2}n^{2}f$ , radiative decay rate (*k*_*r*_) is positively correlated with oscillator strength (*f*) of the pertinent a single photon–atom (or photon–molecule) interaction. For the electronic transition, where $\tilde{v}$ stands for transition frequency in the unit of wavenumber and *n* represents the refractive index of the measured material [71, 72]. The excited state with larger oscillator strength tends to exhibit a larger radiative decay rate. Specifically, for the emission processes of **2**, under two-photon excitation, the final state is mainly dominated by S23 and S24, involved in intermolecular interactions, which are differentiated from the final states S19 and S20 of single-photon localized transition. Taking advantage of the powerful excitation source such as femtosecond laser, high concentration of excited molecules and population of highly excited electronic states could be effectively obtained under two-photon excitation [73]. Therefore, the gigantic ‘blue-shifted’ 2PPL of **2** could be observed due to the radiation from the highly located electronic state. Moreover, the non-radiative decay rate of the highly located excited state is determined by the inter- and intra-molecule vibrational relaxation. The existence of DMF molecules in the 7-fold interpenetrated **1** was due to the larger porosity and may cause the strengthened vibration and rotation of the molecules, which minimized the blue shift of TPPL (*λ*_max_ = 454 nm) in **1**. This observation further confirmed the excitation mechanism of the highly located electronic state under two-photon excitation.

## 3 Conclusions

Gigantic blue-shifted 2PPL emissions in interpenetrated MOFs are an exciting phenomenon. There are very few reports in the literature. [Zn(pvb)_2_]·DMF (**1**) and [Zn(pvb)_2_] (**2**) with 7-fold and 8-fold interpenetrations, respectively, were successfully synthesized. Systematic characterization of emission properties and the corresponding dynamics of Zn (II) MOFs have been revealed and visualized via steady state PL and TRES spectroscopy. A gigantic blue-shifted (∼40 nm) 2PPL emission of 8-fold Zn MOFs and a minor blue shift (∼4 nm) 2PPL of 7-fold interpenetrated MOFs were observed in comparison with their respective PL emissions. The DFT and TDDFT simulations of both one- and two-photon transitions show good agreement with the experimental data and corroborate the observed blue shifts of 2PPL of **2**. The distinctive optical selection rules lead to different final transition states in 1 PA and 2 PA. Under 2 PE, electrons and holes can be more delocalized, and intermolecular interactions mainly govern the emission process of **2** compared to the 1 PA process dominated by LE transition. The calculations reveal rich interplay among the ground, intermediated and final states of the interpenetrated MOFs. Highly excited electronic states of the interpenetrated MOFs were effectively excited and efficiently emitted under femtosecond two-photon excitation, thus generating the inevitable blue-shifted 2PPL emissions. Our work provides a new perspective for exploring the excitation mechanism of fluorescent MOFs and confirms that the interpenetration variation can be used to regulate the nonlinear optical behaviour of MOFs further.

## 4 Experimental section

All the chemicals were of reagent grade and were used without further purification.

### 4.1 Synthesis of trans-2-(4-pyridyl)-4-vinylbenzoic acid (pvb)

The acid Hpvb was prepared by a modified literature method by reacting 100 mmol (9.8 mL) of 4-methylpyridine (4-picoline) with 100 mmol (15.2 g) of 4-formylbenzoic acid in 100 mL acetic anhydride, the reaction mixture was heated at 145 °C for 10 h. The mixture then cooled to room temperature and the precipitated white-yellow crude product was collected by filtration and was washed several times with water followed by ethanol and finally with diethyl ether and recrystallized with hot DMF (16.5 g, yield 75 %).

### 4.2 Preparation of [Zn(pvb)_2_]·DMF (1)

Hpvb (0.022 g, 0.1 mmol) was dispersed in DMF and added a solution of Zn(ClO_4_)_2_·6H_2_O (0.018 g, 0.05 mmol) in H_2_O. The resulting solution was heated in a scintillation vial at 90 °C for 12 h and cooled slowly to room temperature at a rate of 5 °C min^−1^ that gave pale yellow block-shaped single crystals. Yield = 57 %.

### 4.3 Synthesis of [Zn(pvb)_2_] (2)

Hpvb (0.022 g, 0.1 mmol) was dispersed in DMA and added a solution of Zn(ClO_4_)_2_·6H_2_O (0.018 g, 0.05 mmol) in H_2_O. The resulting solution was heated in a scintillation vial at 120 °C for 60 h and cooled slowly to room temperature at a rate of 5 °C min^−1^ that gave thin platy single crystals. Yield = 45 %.

### 4.4 Optical measurements

Absorption spectra were measured by using a Shimadzu UV-3600 spectrometer. Photoluminescence measurements were performed using a 340 nm fs laser pulse. The excitation source is a mode-locked Ti:sapphire laser (Chameleon Ultra II, Coherent) working with repetition rate of 80 MHz, pulse duration of 140 fs. The second harmonic generation of 680 nm output from the laser was employed to excite the samples. The laser beam was focused by a 10× objective lens (N.A. = 0.30) with a radius of ∼1 µm. The PL signals were collected in reflection geometry via an inverted microscope (Nikon Eclipse Ti). Emission from the sample were collected with the same objective lens and routed via a bundled optical fibre to a monochromator (Acton, Spectra Pro 2300i) coupled with a CCD (Princeton Instruments, Pixis 100B). There was no observed sample damage by the laser during the measurements. Time-resolved photoluminescence (TRPL) was detected by using a photon counting photomultiplier (PMT) (PicoQuant, PMA 182), and the signals were processed by using PicoHarp 300. Quantum yield of Zn(II) MOFs were characterized by Edinburg fls920.

Two-photon photoluminescence (2PPL) measurements: the laser beam (680 nm) with a pulse duration of ∼140 fs and a repetition rate of 80 MHz from a femtosecond mode-locked Ti: sapphire laser (Chameleon Ultra II, Coherent) acted as the excitation source. The laser beam was first cleaned by passing through a long pass filter and then reflected by a 50/50 beam splitter into the objective lens (10×, NA = 0.3) to focus onto the sample. The same objective lens was utilized to collect the emission signals. Their spectra were measured by a monochromator (Acton, Spectra Pro 2300i)-coupled CCD (Princeton Instruments, Pixis 100B).

### 4.5 Time-resolved emission spectrum (TRES)

The emitted photons are collected with a photon counting photomultiplier (PMT) (PicoQuant, PMA 182), and the signals were processed by using PicoHarp 300. TRES could be obtained by using the tres-mode when the monochromator can be controlled by PicoHarp 300 software. There was no observed sample damage by the laser during the measurements.

## Supplementary Material

Supplementary Material Details
