# Supplementary material for: Gigantic blue shift of two-photon–induced photoluminescence of interpenetrated metal–organic framework (MOF)
Source: Nanophotonics. 2023 Sep 12;12(19):3781–91. doi: 10.1515/nanoph-2023-0383 (PMC11635949; doi:10.1515/nanoph-2023-0383)
Supplement: Supplementary file 1 — Supplementary Material Details [file j_nanoph-2023-0383_suppl_001.pdf]

Supplementary Information

Gigantic blue shift of two-photon-induced photoluminescence of interpenetrated metal-organic framework (MOF)

Zhihui Chen, Defeng Xu, Menglong Zhu, Yueting Wang, Junfan Feng, Chuancun Shu, Si Xiao,* Jianqiao Meng* and Jun He*

Corresponding author: Si Xiao, Jianqiao Meng and Jun He, Hunan Key Laboratory of Nanophotonics and Devices, School of Physics and Electronics, Central South University, Changsha 410083, China
E-mail: sixiao@csu.edu.cn (S. Xiao), jqmeng@csu.edu.cn (J. Meng) , junhe@csu.edu.cn (J. He)
Zhihui Chen, Defeng Xu, Yueting Wang, Junfan Feng, Chuancun Shu, Hunan Key Laboratory of Nanophotonics and Devices, School of Physics and Electronics, Central South University, Chang-sha 410083, China
Menglong Zhu, Department of Applied Physics, School of Microelectronics and Physics, Hunan University of Technology and Business, Changsha 410205, China

Sample characterization

X-ray powder diffraction (XRPD) measurements

PXRD measurements were performed in transmission mode with a Bruker D8 Advance A25 diffractometer equipped with graphite monochromated Cu Kα radiation (λ = 1.54073 Å) at room temperature.

Thermogravimetric analyses (TGA)

TGA were recorded in a TA Instruments TGA-Q50 thermogravimetric analyzer. Samples were heated at a constant rate of 5ºC min^{-1} from room temperature to 700ºC in a continuous-flow nitrogen atmosphere.

Computational Methods

All the calculations including the geometry optimizations and vibration frequency calculations were carried out in Gaussian16 package[1], and meanwhile, the density functional theory (DFT)[2] and time-dependent density functional theory (TDDFT)[3]were employed for the calculations of the ground states and singlet excited states, respectively. For the geometry optimizations of the ground states and singlet excited states, the restricted CAM-B3LYP method[4] with cc-PVTZ basis set[5] for the Zn atom and 6-31+G(d, p) basis set[6] for other atoms were employed. The vibration frequency calculations were carried out at the same computational method and basis sets to make sure that the optimized structures were true energy minima, and meanwhile, obtain the absolute free energies of the optimized structures.

The optimized structures of ground states were further employed to process the electron excitation calculations, and a total of 50 excited states were considered. According to the excited energy (v_i) and oscillator strength (f_i) for each excited state obtained by the electron excitation calculations, the calculated UV absorption spectrums (i.e., one-proton absorption (OPA) spectrums) were obtained by the following formula (**Equation 1**):

$$G(v) = \sum_i \left[1.619602 * 10^4 * \frac{f_i}{\sigma} * e^{-(\frac{v-v_i}{\sigma})^2}\right] \qquad (\textbf{Equation 1})$$

where $2\sigma(\ln 2)^{1/2}$ was equal to the full width half maximum (FWHM) of the spectrum curves, and in our curves 0.66667ev was adopted. And the calculated *G(v)* values (unit: L/mol/cm) and wavelength values (v, unit: ev, could be converted to wavelength unit: nm=1239.842/ev) were employed to draw the one-photon absorption (OPA) spectrums.

Besides the excited energy (v_i) and oscillator strength (f_i) for each excited state, the transition dipole moments between ground state and excited states as well as between excited states, and the energies of excited states, also obtained from the electron excitation calculations were employed to further calculate the two-photon absorption (TPA) cross section (δ_{tp}) by following the below formula (**Equation 2**)[7–11]:

$$\delta_{tp} = 8 \sum_{\substack{j\neq g \\ j\neq f}} \left[\frac{|<f|\mu|j><j|\mu|g>|^2}{\left(\omega_j - \frac{\omega_f}{2}\right)^2 + \tau_f^2} (1 + 2cos^2\theta_j) \right] + 8 \left[\frac{\left|\Delta\mu_{fg}\right|^2 |<f|\mu|g>|^2}{\left(\frac{\omega_f}{2}\right)^2 + \tau_f^2} (1 + 2cos^2\emptyset) \right] \qquad (\textbf{Equation 2})$$

where the first term and second term of this formula were the three-state model which was used to describe the transition process from the ground state to the intermediate state, and the two-state model which was used to described the transition process from the intermediate state to the final state, respectively. The $|g>$, $|j>$, $|f>$ and μ were the ground state wave function, intermediate wave function, final state wave function and transition dipole moment, respectively. Therefore, it was not hard to see that the $<j|\mu|g>$, $<f|\mu|j>$ and $<f|\mu|g>$ were the vectors of the transition dipole moments from the ground state to the intermediate state, from the intermediate state to the final state, as well as from the ground state to the final state, respectively. Meanwhile, $\Delta\mu_{fg}$ was the difference

between the permanent dipole moment vectors of final state and ground state. In other words, $\Delta\mu_{fg}$ = <$f|\mu|f$> - <$g|\mu|g$>. Correspondingly, the θ_j was the vector angle between the transition dipole moment vectors of <$j|\mu|g$> and <$f|\mu|j$>, and the ϕ was the vector angle between the vector of $\Delta\mu_{fg}$ and the transition dipole moment vector of <$f|\mu|g$>. In addition, the ω_j and ω_f were the excited energies of intermediate states and final states, respectively. Finally, the τ_f was the lifetime of final state, and the excited energy (v_i) and oscillator strength (f_i) were employed to calculated the lifetime by following the below formula (**Equation 3**):

$$\tau_f = \frac{1.499194}{v_i^2 * f_i} \qquad (\boldsymbol{Equation\ 3})$$

where the units of life (τ_f) and excited energy (v_i) were time (unit: s) and wavenumber (unit: cm^{-1}, could be converted from ev unit: cm^{-1} = 8065.5447 * ev), respectively.

Based on the calculated two-photon absorption (TPA) cross section (δ_{tp}) for each excitation from ground state to final state obtained from the Equation 2, the Equation 1 was once again employed to generate the two-photon absorption (TPA) spectrums. And during the generation process, the two-photon absorption (TPA) cross section (δ_{tp}) referred to the oscillator strength (f_i). And the final calculated $G(v)$ values (unit: L/mol/cm) and wavelength values (v, unit: ev, could be converted to wavelength unit: nm=1239.842/ev) were employed to draw the two-photon absorption (TPA) spectrums.

During the optimization processes of the singlet excited states, the electron excitation calculations were also considered by default, and the output excited energy (v_i) and oscillator strength (f_i) of the corresponding optimized states were adopted to generated the emission spectrum also by employing the Equation 1. And the calculated $G(v)$ values (unit: L/mol/cm) and wavelength values (v, unit: ev, could be converted to wavelength unit: nm=1239.842/ev) were employed to draw the emission spectrums. Above processes of generating absorption spectrums and emission spectrums have realized the automation by using a python script wrote by ourselves.

In addition to the absorption spectrums and emission spectrums, the charge transfer matrix (CTM) analysis from ground state to excited state, the electron-hole analysis from ground state to excited state, the transition density matrix (TDM) between excited states, as well as the differential charge density (DCD) analysis between excited states were constructed to clarify the differences between the processes of the one-proton

excitation and the two-proton excitation. The output results of electron excitation calculations were employed to generate above analysis results by using the Multiwfn 3.8 program[12].

Supporting Figures

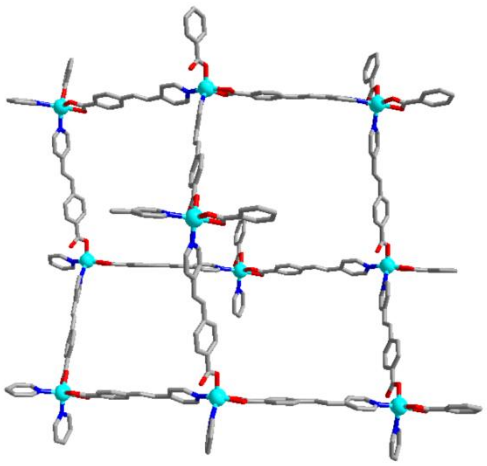

Figure S1. Individual diamondoid connectivity in **1**.

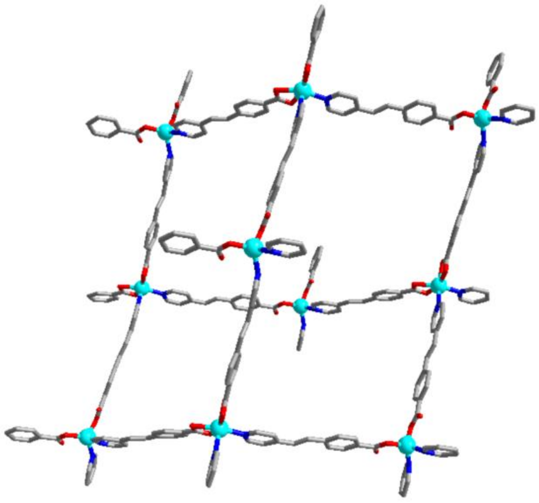

Figure S2. Individual diamondoid connectivity in **2.**

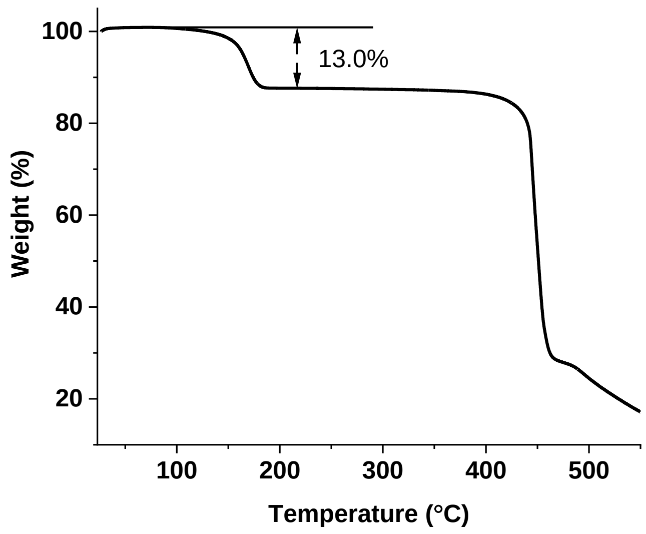

Figure S3. TG of [Zn(pvb)$_2$] ·DMF, **1** Thermal analysis shows a weight loss of 13.0% from RT to 210°C, corresponding to loss of one DMF molecule (calculated, 12.5%).

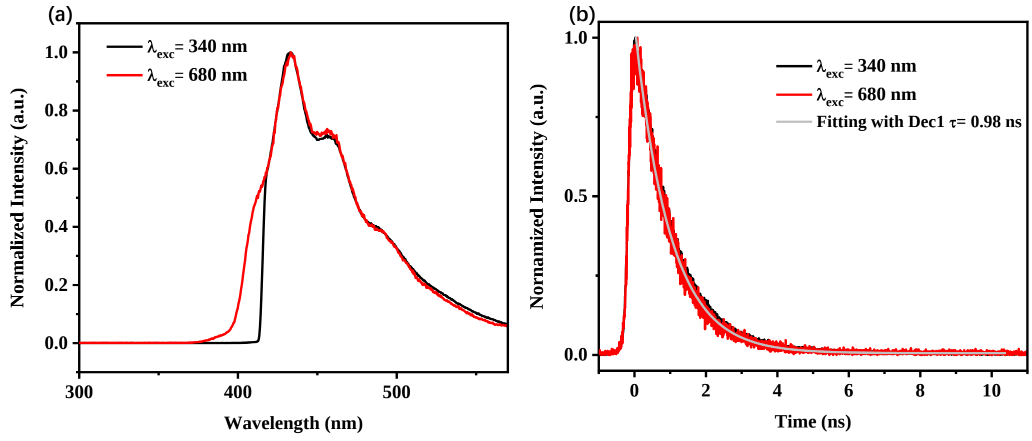

Figure S4. PL spectra and time-resolved photoluminescence (TRPL) of Hpvb ligand. The emission dynamics of Hpvb ligand were similar for one-photon and two-photon excitation situations. TRPL of Hpvb ligand was fit with a single exponential decay with $\tau_{Hpvb} = 0.98\,ns$.

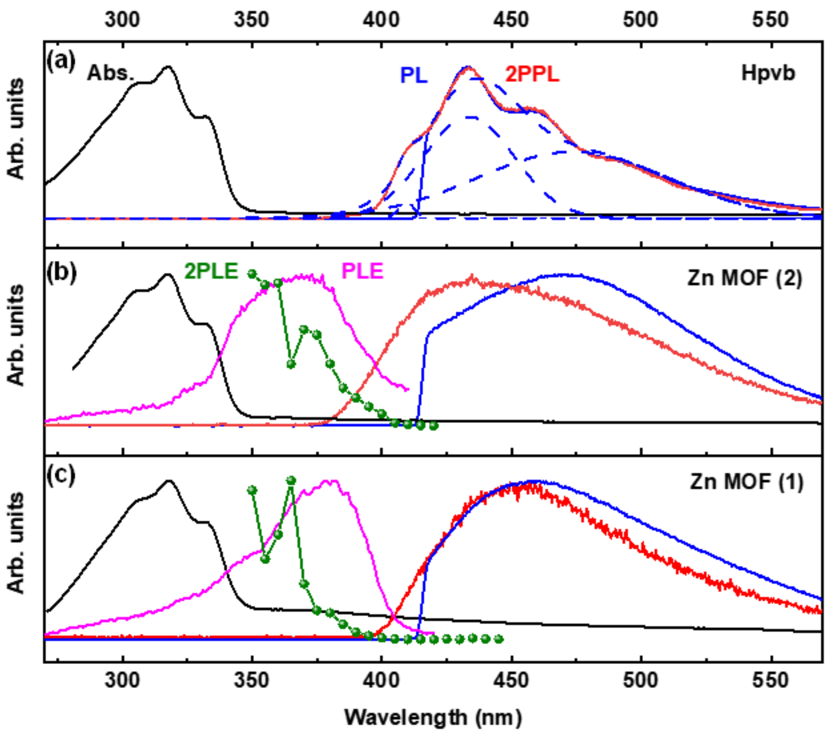

Figure S5. Normalized absorption (black line), PL under 340 nm excitation (blue line), 2PPL under 680 nm excitation (red line), PLE (pink line) and 2PLE (olive symbol-line) of Hpvb ligand (a), 2 (b) and 1 (c). Emission peak analysis of Hpvb by Gaussian function (dash blue line).

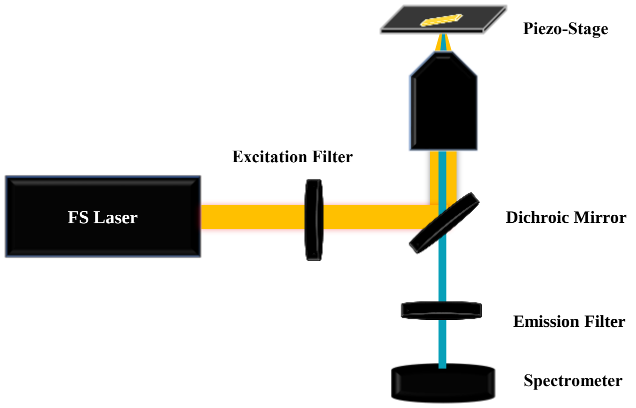

Figure S6. Experimental setup for multiphoton-excited PL on the crystallites.

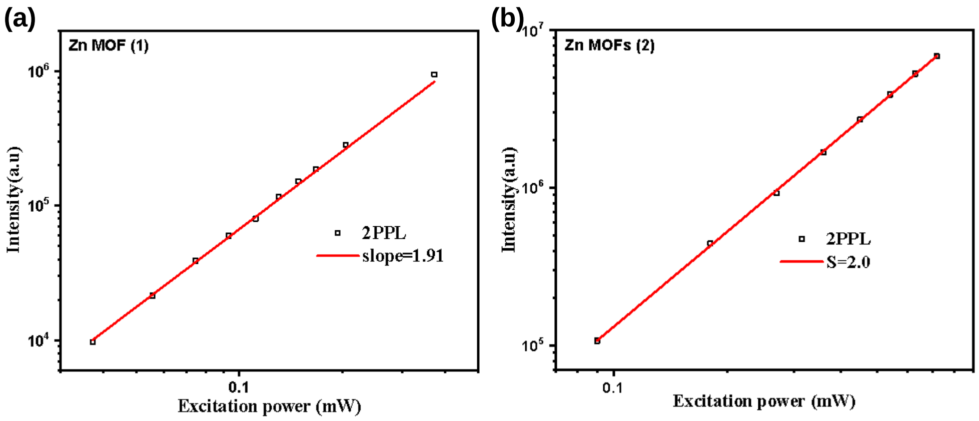

Figure S7. Log−log plots for the integrated 2PP signals vs the excitation laser power for **1** (a) and **2** (b) under 680 nm excitation.

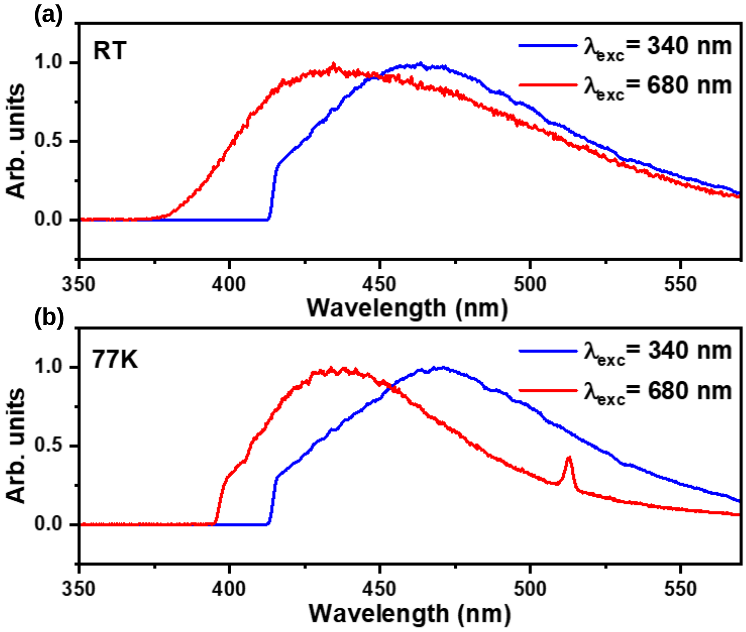

Figure S8. Steady-state PL ($\lambda_{exc} = 340\ nm$) and 2PPL ($\lambda_{exc} = 680\ nm$) spectra of **2** was conducted under RT (a) and 77K (b). The small peak located at 512 nm in the 2PPL spectrum under 77K was generated by the substrate.

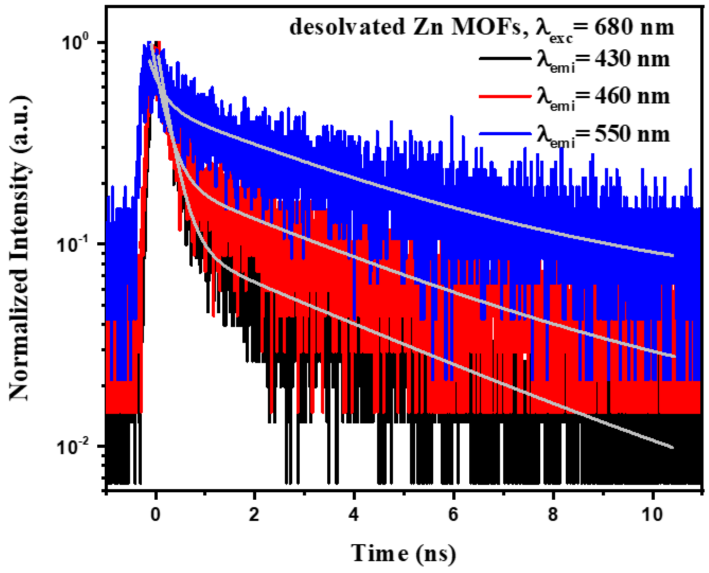

Figure S9. TRPL curves of **2** under two-photon excitation ($\lambda_{exc} = 680\ nm$). The dynamic processes of 2PPL of **2** were fit with biexponential decays. As the collection wavelength increases from 430 nm to 550 nm, the corresponding PL lifetime expands from ~ 2.70 ns to ~ 3.98 ns.

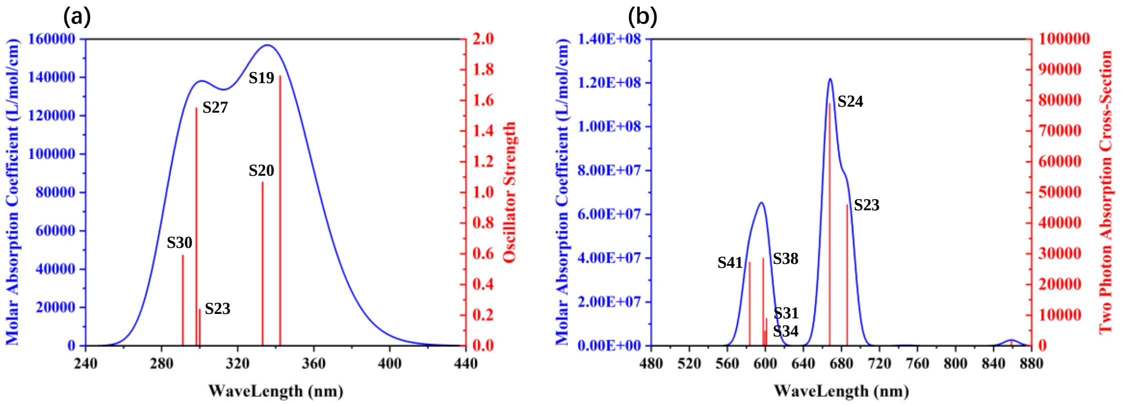

Figure S10. (a) Calculated One-photon absorption (1PA) spectrum and (b) two-photon absorption (2PA) spectrum of **2**.

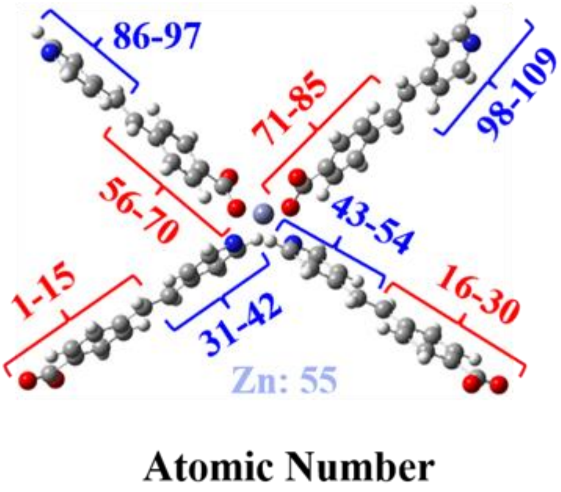

Figure S11. The atomic numbers of **2**. In the following charge transfer matrix (CTM) maps and transition density matrix (TDM) maps, their x-axis and y-axis denoted these atomic numbers.

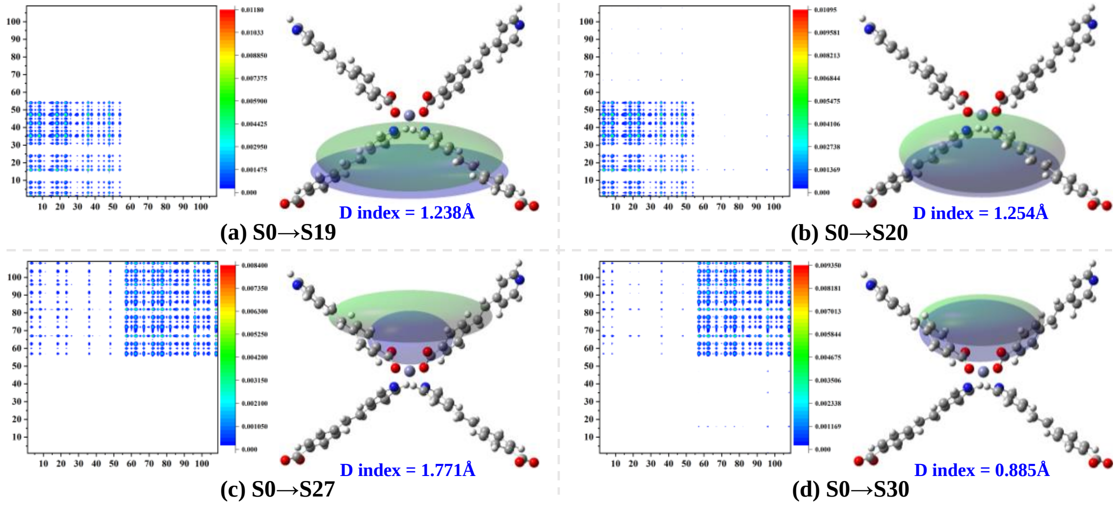

Figure S12. Charge transfer matrix (CTM) maps and distribution of electron (colored by green ellipse) and hole (colored by blue ellipse) of (a) S0→S19 transition, (b) S0→S20 transition, (c) S0→S27 transition, and (d) S0→S30 transition. The x-axis and y-axis in the charge transfer matrix (CTM) maps denoted the atomic numbers in Figure S8. The D index was used to evaluate the separation degree of the electron and hole.

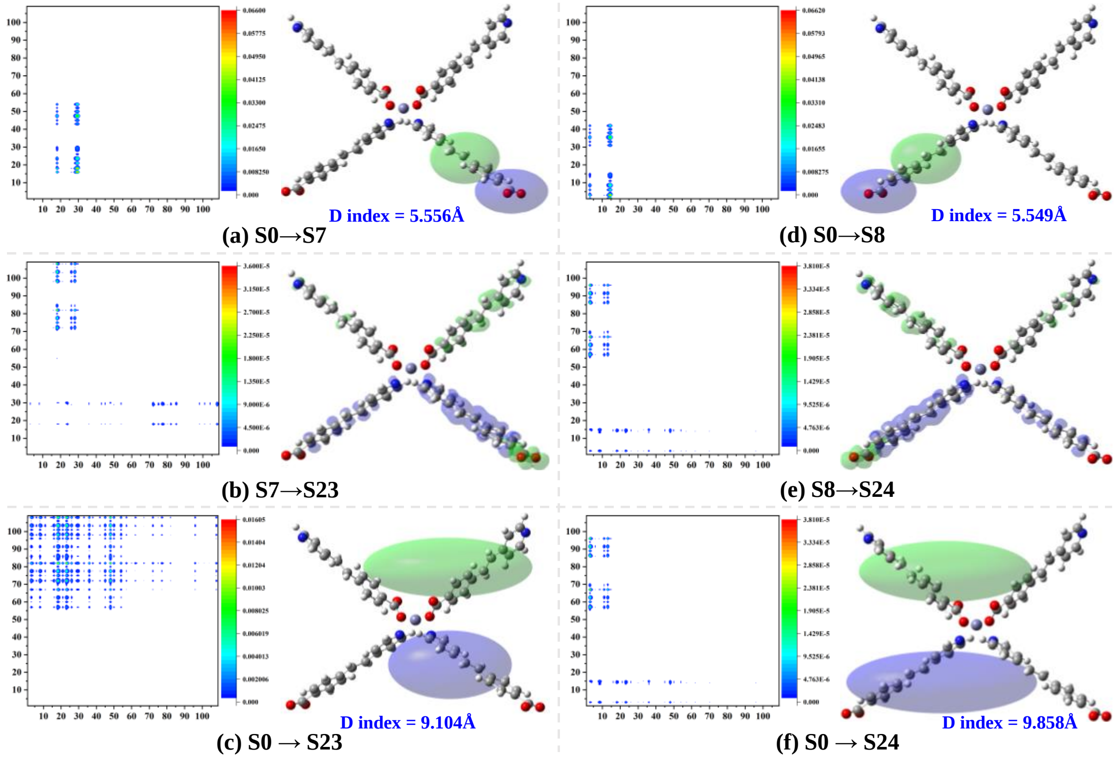

Figure S13. Charge transfer matrix (CTM) maps and distribution of electron (colored by green ellipse) and hole (colored by blue ellipse) of (a) S0→S7 transition, (c) S0→S23 transition, (d) S0→S8 transition, and (f) S0→S24 transition as well as transition density matrix (TDM) maps and differential charge density (DCD) maps of (b) S7→S23 transition and (e) S8→S24 transition. The x-axis and y-axis in the charge transfer matrix (CTM) maps and transition density matrix (DCD) maps denoted the atomic numbers in Figure S8. The D index was used to evaluate the separation degree of the electron and hole. Meanwhile, in the differential charge density (DCD) maps, green and blue represented the regions where electron density increased and decreased during transition process, respectively.

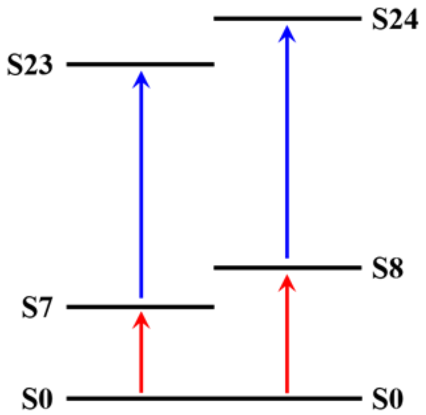

Figure S14. Two main transition processes involved in 2PA process of **2**.

Supporting Tables

Table S1. The detailed fitting parameters of TRPL curves of **2** upon one- (340 nm) and two-photon (680 nm) excitation.

$\lambda_{exc}(nm)$	$\lambda_{emi}(nm)$	$A_1(\%)$	$\tau_1(ns)$	$A_2(\%)$	$\tau_1(ns)$	$\tau(ns)$
	**430**	**3**	**0.25**	**97**	**4.1**	**3.98**
**340**	**460**	**23**	**0.25**	**77**	**4.1**	**3.21**
	**550**	**51**	**0.25**	**49**	**4.1**	**2.13**
	**430**	**2.7**	**0.25**	**97.3**	**4.1**	**3.98**
**680**	**460**	**14**	**0.25**	**86**	**4.1**	**3.56**
	**550**	**36**	**0.25**	**64**	**4.1**	**2.7**

Table S2. Main transition processes involved in 2PA.

2PA states	Channel	Processes
**S23**	Channel 1	$<\phi S0|\mu|\phi S7> \rightarrow <\phi S7|\mu|\phi S23>$
**S24**	Channel 1	$<\phi S0|\mu|\phi S8> \rightarrow <\phi S8|\mu|\phi S24>$
**S31**	Channel 1	$<\phi S0|\mu|\phi S7> \rightarrow <\phi S7|\mu|\phi S31>$
	Channel 2	$<\phi S0|\mu|\phi S16> \rightarrow <\phi S16|\mu|\phi S31>$
**S34**	Channel 1	$<\phi S0|\mu|\phi S8> \rightarrow <\phi S8|\mu|\phi S34>$
	Channel 2	$<\phi S0|\mu|\phi S18> \rightarrow <\phi S18|\mu|\phi S34>$
**S38**	Channel 1	$<\phi S0|\mu|\phi S7> \rightarrow <\phi S7|\mu|\phi S38>$
	Channel 2	$<\phi S0|\mu|\phi S16> \rightarrow <\phi S16|\mu|\phi S38>$
**S41**	Channel 1	$<\phi S0|\mu|\phi S8> \rightarrow <\phi S8|\mu|\phi S41>$
	Channel 2	$<\phi S0|\mu|\phi S18> \rightarrow <\phi S18|\mu|\phi S41>$

References

[1] M. J. Frisch, G. W. Trucks, H. B. Schlegel, G. E. Scuseria, Ma. Robb, J. R. Cheeseman, G. Scalmani, V. Barone, G. A. Petersson, H. Nakatsuji, *Inc., Wallingford CT* **2016**, *3*.

[2] W. Kohn, L. J. Sham, *Phys. Rev.* **1965**, *140*, A1133.

[3] F. Furche, R. Ahlrichs, *J. Chem. Phys.* **2002**, *117*, 7433.

[4] T. Yanai, D. P. Tew, N. C. Handy, *Chem. Phys. Lett.* **2004**, *393*, 51.

[5] A. K. Wilson, T. van Mourik, T. H. Dunning, *J. Mol. Struct. THEOCHEM* **1996**, *388*, 339.

[6] A. D. McLean, G. S. Chandler, *J. Chem. Phys.***1980**, *72*, 5639.

[7] M. Richter, S. Mukamel, *Phy. Rev. A* **2011**, *83*, 063805.

[8] K. E. Dorfman, F. Schlawin, S. Mukamel, *Rev. Mod. Phys.* **2016**, *88*, 045008.

[9] M. Sun, Y. Ding, L. Zhao, F. Ma, *Chem. Phys.* **2009**, *359*, 166.

[10] M. Sun, J. Chen, H. Xu, *J. Chem. Phys.* **2008**, *128,* 064106.

[11] X. Mu, J. Wang, M. Sun, *J. Phys. Chem. C* **2019**, *123*, 14132.

[12] T. Lu, F. Chen, *J. Comput. Chem.* **2012**, *33*, 580.
